# Association of renin–angiotensin system inhibitors use with short- and long-term mortality in patients with aortic stenosis: A systematic review and meta-analysis

**DOI:** 10.3389/fcvm.2022.917064

**Published:** 2023-01-23

**Authors:** Yang Guan, Xiangyun Kong, Huagang Zhu, Hong Li, Lihan Zhao, Fei Guo, Qiang Lv

**Affiliations:** ^1^Department of Cardiology, Beijing Anzhen Hospital, Capital Medical University, Beijing, China; ^2^Department of General Medicine, Beijing Luhe Hospital, Capital Medical University, Beijing, China

**Keywords:** renin-angiotensin system inhibitors, aortic stenosis, transcatheter aortic valve replacement, surgical aortic valve replacement, short-term mortality, long-term mortality

## Abstract

**Purpose:**

The present study aimed to investigate the association of renin–angiotensin system inhibitors (RASi) with short- and long-term mortality in patients with aortic stenosis (AS).

**Methods:**

A systematic search was performed in PubMed, Embase, and Cochrane library databases for relevant studies published before March 2022. Studies meeting the inclusion criteria were included to assess the effect of RASi on short-term (≤30 days) and long-term (≥1 year) mortality in patients with AS.

**Results:**

A total of 11 studies were included in the meta-analysis. Our results demonstrated that RASi reduced short-term mortality (OR = 0.76, 95% CI 0.63–0.93, *p* = 0.008) after aortic valve replacement (AVR). Subgroup analysis revealed that RASi was still associated with lower short-term mortality after transcatheter aortic valve replacement (TAVR); however, the association was relatively weak in patients who underwent surgical aortic valve replacement (SAVR). For long-term mortality, the pooled OR was 1.04 (95% CI 0.88–1.24, *p* = 0.63) after sensitivity analysis in patients who did not undergo AVR. In addition, our study confirmed that RASi significantly reduced long-term mortality (OR = 0.57, 95% CI 0.44–0.74, *p* < 0.0001) in patients who underwent AVR. Subgroup analysis showed that both TAVR and SAVR groups treated with RASi had lower long-term mortality.

**Conclusion:**

Renin–angiotensin system inhibitors did not change long-term mortality in AS patients who did not undergo AVR. However, RASi reduced short- and long-term mortality in patients who underwent AVR.

## Introduction

Aortic stenosis (AS) is a common primary valve disease in the aging population, affecting 2–7% of subjects older than 65 years (1, 2). AS is a progressive disease without obvious symptoms in the early stages. It is associated with increased cardiovascular morbidity and mortality (3, 4). To date, the appropriate timing of aortic valve replacement (AVR), including surgical aortic valve replacement (SAVR) and transcatheter aortic valve replacement (TAVR), is the main treatment strategy of AS with documented survival benefits (5, 6).

Even in its early stage, AS leads to left ventricular hypertrophy and fibrosis through several mechanisms, one of which is renin–angiotensin system (RAS) activation (7, 8). Severe left ventricular hypertrophy and fibrosis were associated with poor prognosis in patients with AS (9, 10). Herein, treatment with renin–angiotensin system inhibitors (RASi), including angiotensin-converting enzyme inhibitors (ACEi) and angiotensin-receptor blockers (ARBs), attenuates cardiac remodeling and myocardial hypertrophy and fibrosis in patients with AS (11, 12). However, the effect of RASi on prognosis has not been well characterized, and the results of previous studies in this field have been inconsistent (2, 13, 14).

Therefore, this meta-analysis of available randomized and observational studies aimed to explore whether treatment with RASi is associated with short- and long-term mortality in patients with AS.

## Materials and methods

We performed this meta-analysis in accordance with the Cochrane Handbook for Systematic Review of Intervention. In addition, the meta-analysis has been reported according to the Meta-Analysis of Observational Studies in Epidemiology (MOOSE) statement (15). The preferred reporting items for systematic reviews and meta-analyses statement (PRISMA) checklist is showed in Supplementary Table 2.

### Search strategy

A systematic search was performed in PubMed, Embase, and Cochrane library databases among articles published before March 2022. The following Medical Subject Heading (MeSH) terms or free texts were used for searching: renin-angiotensin system inhibitors, angiotensin-converting enzyme inhibitors, angiotensin II receptor blockers, aortic valve stenoses, (stenoses, aortic valve), (stenosis, aortic valve), (valve stenoses, aortic), (valve stenosis, aortic), aortic stenosis, (stenoses, aortic), (stenosis, aortic). We also reviewed the reference lists of retrieved articles for additional studies.

### Eligibility and study selection

Studies meeting the following inclusion criteria were selected: (1) randomized controlled trials, prospective cohort studies, and retrospective cohort studies reporting the effect of RASi on short-term (≤30 days) or long-term (≥1 year) all-cause mortality in patients with AS; (2) provided the number of all-cause death; and (3) performed in adults more than 18 years. Reviews, comments, conference abstracts, and editorials were all excluded.

### Data extraction and quality assessment

Two investigators independently extracted the following information with standardized data abstraction form from eligible studies: first author’s name, publication year, study design, the number of patients, the number of all-cause mortality, baseline characteristics, and echocardiographic data. The quality of the studies included in this meta-analysis was assessed by two independent investigators using the 9-star Newcastle–Ottawa Scale (NOS) and Revised Jadad’s Scale. Any discrepancies regarding data extraction and quality assessment were resolved by discussing with a third researcher under the supervision of the senior researcher.

### Statistical analysis

The overall odd ratios (ORs) with 95% confidence intervals (CIs) for all-cause mortality were calculated using the reported number of all-cause death in the RASi group and non-RASi group in eligible studies. We calculated the trial-specific ORs using a fixed- or random-effects model depending on the heterogeneity of data included in this meta-analysis. The heterogeneity of data was evaluated by Cochran’s *Q* test and *I*^2^ statistic. The fixed-effects model was used in the absence of significant heterogeneity (*p* > 0.1, *I*^2^ < 50%) among eligible studies. In the presence of significant heterogeneity (*p* < 0.1, *I*^2^ ≥ 50%) among studies, a sensitivity analysis or subgroup analysis was performed to explore the sources of heterogeneity. The funnel plot was not performed due to the limited number of studies included in the present meta-analysis. The pooled analyses in the present study were performed using Review Manager, version 5.3 (Cochrane Collaboration, Copenhagen, Denmark). All tests were 2-tailed, and *p*-values were considered statistically significant at <0.05.

## Results

### Study selection and quality assessment

**FIGURE 1:**
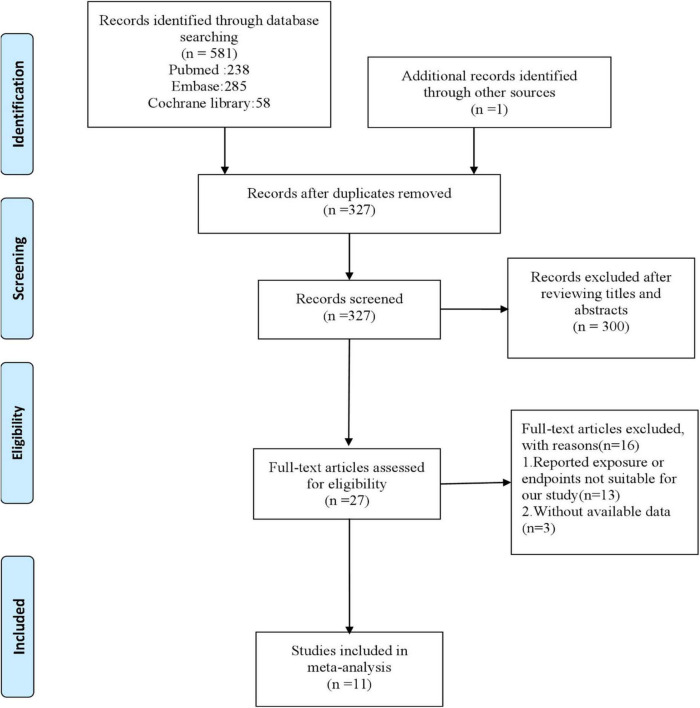
Flowchart of study search and identification.

In total, 581 articles were identified by a systematic search in PubMed, Embase, and Cochrane library databases, and 1 article was identified from reference lists of the eligible studies. After removing duplicates and excluding non-relevant articles, 27 articles remained for full-text assessment. According to the inclusion criteria, 11 studies were used in this meta-analysis (2, 11–14, 16–21). The detailed flow diagram of this study is presented in Figure 1.

The quality of eligible studies assessed by NOS and Revised Jadad’s Scale is shown in Supplementary Table 1. The mean NOS score was 8 stars, and Revised Jadad’s score was 4, indicating satisfactory study quality.

### Characteristics of eligible studies and populations

As can be seen in Table 1, 10 of 11 studies included in this meta-analysis were cohort studies (2, 12–14, 16–21) and 1 of 11 was a randomized controlled trial (11). These 11 eligible studies consisted of 33,858 patients with AS, including 16,299 RASi users and 17,559 non-RASi users. The range follow-up length varied from 1 to 8 years.

**TABLE 1:** Characteristics of included studies.

References	Design	Multicenter	Number of patients		Mean or median follow-up time (years)
			RASi group	Non-RASi group	
Dahl et al. (11)	Randomized controlled trial	No	57	57	1
Nadir et al. (14)	Retrospective cohort study	No	699	1418	4.2
Ardehali et al. (13)	Retrospective cohort study	No	2017	2088	1
Goel et al. (20)	Retrospective cohort study	No	741	1011	5.8
Bang et al. (2)	Retrospective cohort study	Yes	769	1104	4.3
Ochiai et al. (12)	Prospective cohort study	Yes	371	189	2
Magne et al. (19)	Retrospective cohort study	No	268	240	4.8
Inohara et al. (17)	Retrospective cohort study	Yes	7948	7948	1
Klinkhammer (18)	Retrospective cohort study	No	71	98	1
Rodriguez-Gabella et al. (21)	Retrospective cohort study	Yes	1622	1163	3
Chen et al. (16)	Retrospective cohort study	Yes	1736	2243	2

RASi, renin–angiotensin system inhibitors.

The demographic, clinical, and echocardiographic data of patients are presented in Tables 2, 3. The mean age of patients ranged from 67.2 to 84.8 years and more than half were male. In addition, more than half of the patients had hypertension, and the majority of patients had preserved ejection fraction.

**TABLE 2:** Demographic and clinical characteristics.

References	Age (years)		Gender % (male)		Hypertension %		DM %		CAD %	
	RASi	Non-RASi	RASi	Non-RASi	RASi	Non-RASi	RASi	Non-RASi	RASi	Non-RASi
Dahl et al. (11)	72.3 ± 8	72.6 ± 10	59	65	42	41	19	12	21	16
Nadir et al. (14)	73 ± 10	74 ± 13	49	45	NA	NA	28	12	NA	NA
Ardehali et al. (13)	72	71	98	96	92	57	49	18%	NA	NA
Goel et al. (20)	72 ± 9	72 ± 10	60.3	61	84.5	63.6	29.7	19.1	58.9	52.4
Bang et al. (2)	68.0 ± 9.1	67.2 ± 9.9	61	62	79	21%	NA	NA	NA	NA
Ochiai et al. (12)	84.2 ± 5.0	84.8 ± 5.0	29.9	24.3	83.8	61.4	27.8	24.3	41.5	33.9
Magne et al. (19)	74 ± 10	74 ± 9	55	56	91	91	16	21	22	24
Inohara et al. (17)	82.4 ± 6.8	82.4 ± 6.9	52.3	51.6	93.6	93.1	38.7	38.7	NA	NA
Klinkhammer (18)	77.8 ± 7.9	80.1 ± 7.5	56	61	96%	77	51	32	75	77
Rodriguez-Gabella et al. (21)	80.8 ± 7.01	80.7 ± 7.18	45.1	46.9	85.6	74.8	36.4	31.7	39.1	32.9
Chen et al. (16)	81.7 ± 7.2	82.9 ± 7.7	60.8	58.4	96.3	89.7	40.6	32.5	81.0	76.0

RASi, renin–angiotensin system inhibitors; DM, diabetes mellitus; CAD, coronary artery disease; NA, not applicable.

**TABLE 3:** Echocardiographic data.

References	LVEF %		PAJV (m/s)		Aortic valve area (cm^2^)		Mean gradient (mmHg)		LVMI (g/m^2^)	
	RASi	Non-RASi	RASi	Non-RASi	RASi	Non-RASi	RASi	Non-RASi	RASi	Non-RASi
Dahl et al. (11)	54 ± 7	54 ± 8	3.9 ± 0.9	3.9 ± 0.7	0.81 ± 0.29	0.81 ± 0.27	NA	NA	137 ± 48	130 ± 32
Nadir et al. (14).	LVSD (14)	LVSD (11)	3.3 ± 0.9	3.5 ± 0.7	1.0 ± 0.4	0.9 ± 0.4	30 ± 17	38 ± 19	NA	NA
Ardehali et al. (13)	52	54	NA	NA	NA	NA	NA	NA	NA	NA
Goel et al. (20)	LVSD (22.1)	LVSD (16.4)	NA	NA	0.69 ± 0.13	0.66 ± 0.14	46 ± 15	49 ± 17	128 ± 39	128 ± 40
Bang et al. (2)	65 ± 9	66 ± 8	3.1 ± 0.6	3.1 ± 0.5	NA	NA	NA	NA	104 ± 33	97 ± 29
Ochiai et al. (12)	62.9 ± 13.1	63.3 ± 11.9	4.6 ± 0.8	4.6 ± 0.7	0.64 ± 0.17	0.62 ± 0.16	50.8 ± 18.4	50.6 ± 16.8	136 ± 36	125 ± 39
Magne et al. (19)	66 ± 13	65 ± 12	NA	NA	0.68 ± 0.1	0.70 ± 0.2	53 ± 16	49 ± 17	NA	NA
Inohara et al. (17)	51.9 ± 11.5	52.0 ± 11.5	NA	NA	NA	NA	NA	NA	NA	NA
Klinkhammer (18)	55.6 ± 14.1	58.6 ± 11.6	4.12 ± 0.63	4.08 ± 0.62	0.96 ± 0.34	0.91 ± 0.25	44.2 ± 13.3	43.5 ± 12.1	NA	NA
Rodriguez-Gabella et al. (21)	57.4 ± 13.9	58.9 ± 13.4	NA	NA	0.68 ± 0.19	0.65 ± 0.18	47.3 ± 15.7	48.9 ± 16.6	NA	NA
Chen et al. (16)	54.5 ± 13.7	54.9 ± 13.3	NA	NA	0.69 ± 0.18	0.68 ± 0.18	44.7 ± 13.0	44.5 ± 13.5	123.4 ± 33.5	122.1 ± 34.4

RASi, renin–angiotensin system inhibitors; LVEF, left ventricular ejection fraction; PAJV, peak aortic jet velocity; LVMI, left ventricular mass index, LVSD, left ventricular systolic dysfunction; NA, not applicable.

### Effect of RASi on short-term mortality

**FIGURE 2:**
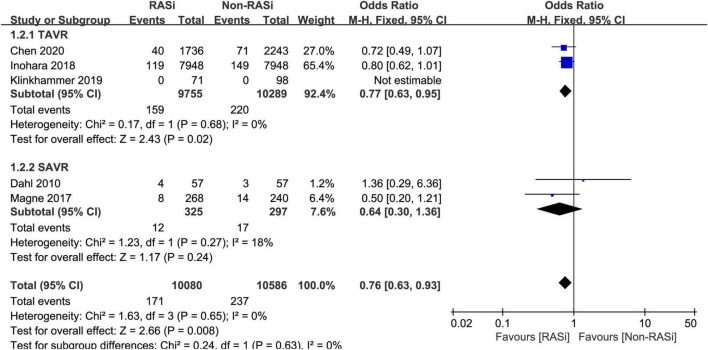
Forest plot comparing short-term mortality between patients with RASi-treated and non-RASi-treated AS who underwent AVR. RASi, renin–angiotensin system inhibitors; AS, aortic stenosis; AVR, aortic valve replacement. SAVR, surgical aortic valve replacement; TAVR, transcatheter aortic valve replacement.

Five studies comprising 20,666 patients with AS reported the effect of RASi on short-term mortality (11, 16–19). However, the studies were all conducted in patients with AS who underwent AVR. After pooling data from 10,800 RASi users and 10,586 non-RASi users by the fixed-effects model (*p* = 0.65, *I*^2^ = 0%), we found that the RASi group had a lower risk of short-term mortality (OR = 0.76, 95% CI 0.63–0.93, *p* = 0.008, Figure 2) after AVR.

Subsequently, we performed a subgroup analysis to investigate the effect of RASi on short-term mortality in patients who underwent SAVR (11, 19) and TAVR (16–18). In patients with AS, receiving RASi was associated with a lower risk of mortality (OR = 0.77, 95% CI 0.63–0.95, *p* = 0.02, Figure 2) within 30 days after TAVR. Among patients who underwent SAVR, the RASi group also had lower 30-day mortality (OR = 0.64, 95% CI 0.30 to 1.36, *p* = 0.24, Figure 2); however, it was not statistically significant.

### Effect of RASi on long-term mortality

**FIGURE 3:**
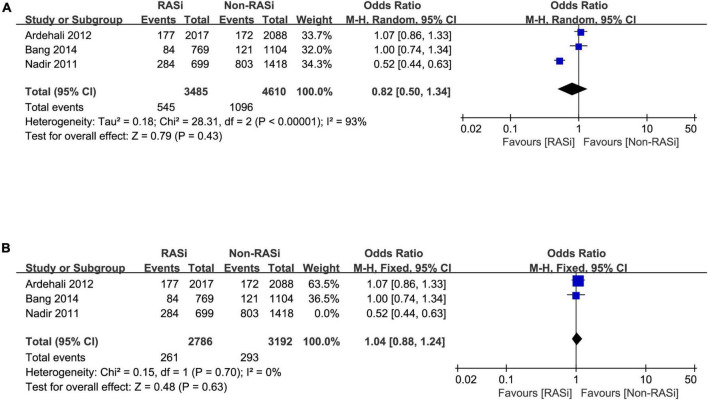
Forest plot comparing long-term mortality between patients with RASi-treated and non-RASi-treated AS who did not undergo AVR **(A)**. Forest plot comparing long-term mortality between patients with RASi-treated and non-RASi-treated AS who did not undergo AVR after sensitivity analysis **(B)**. RASi, renin–angiotensin system inhibitors; AS, aortic stenosis; AVR, aortic valve replacement.

Three studies reported the effect of RASi on long-term mortality in patients with AS who did not undergo AVR (2, 13, 14). After pooling the 3 studies with a random-effects model, we found that there is no increased risk of long-term mortality in patients with AS treated with RASi (OR = 0.82, 95% CI 0.50–1.34, *p* = 0.43, Figure 3A). Due to the remarkable heterogeneity (*p* < 0.00001, *I*^2^ = 93%), a sensitivity analysis was performed. After excluding the study causing the heterogeneity (14), our pooled results still demonstrated that RASi did not increase the risk of long-term mortality (OR = 1.04, 95% CI 0.88–1.24, *p* = 0.63, Figure 3B).

**FIGURE 4:**
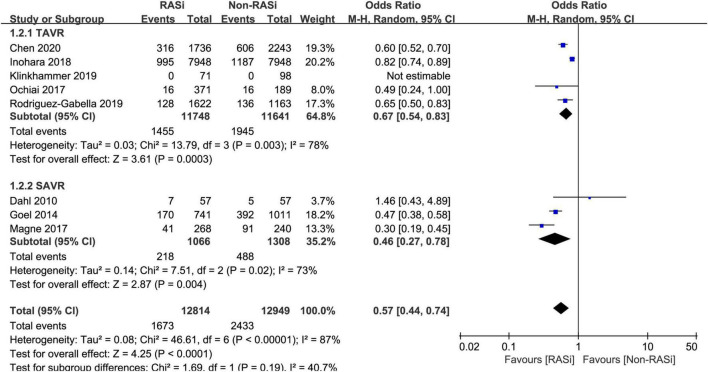
Forest plot comparing long-term mortality between patients with RASi-treated and non-RASi-treated AS who underwent AVR. RASi, renin–angiotensin system inhibitors; AS, aortic stenosis; AVR, aortic valve replacement. SAVR, surgical aortic valve replacement; TAVR, transcatheter aortic valve replacement.

As can be seen in Figure 4, our results also confirmed that treatment with RASi was associated with lower long-term mortality (OR = 0.57, 95% CI 0.44–0.74, *p* < 0.0001) in patients who underwent AVR, after pooling 8 studies with 25,763 patients by a random-effects model (11, 12, 16–21).

Meanwhile, a subgroup analysis was conducted to investigate the effect of RASi on long-term mortality in patients who underwent SAVR (11, 19, 20) and TAVR (12, 16–18, 21). Our pooled analysis indicated that treatment with RASi in patients who underwent TAVR was significantly associated with lower long-term mortality (OR = 0.67, 95% CI 0.54–0.83, *p* = 0.0003, Figure 4). Among patients who underwent SAVR, the negative association still persisted (OR = 0.46, 95% CI 0.27–0.78, *p* = 0.004, Figure 4).

### Publication bias and sensitivity analysis

Based on a visual inspection of funnel plots (Supplementary Figures 1–3), there was no obvious evidence of publication bias among the included studies. In addition, we performed a sensitivity analysis for long- or short-term all-cause mortality and found that the pooled results remained stable after the sensitivity analysis.

## Discussion

This meta-analysis of 11 clinical trials had three significant findings. First, treatment with RASi reduced short-term mortality in patients who underwent AVR. Subgroup analysis demonstrated that the association persisted in patients with AS who were treated with TAVR. Second, RASi was safe for patients with AS who did not undergo AVR and did not increase long-term mortality. Third, among patients who underwent AVR, using RASi was associated with a lower risk of long-term mortality. After subgroup analysis, the association still persisted in patients with AS who underwent TAVR or SAVR.

Previously, treatment with drugs like RASi was considered unsafe and even contraindicated in patients with significant AS (22, 23). These drugs were assumed to induce severe hypotension due to the vasodilator induced by them and the fixed obstruction at left ventricular outflow in patients with AS (22). Although this risk has never been confirmed by clinical evidence, many studies have suggested that treatment with RASi is well tolerated in patients with mild-to-severe AS (24, 25). In addition, some studies even reported the beneficial effects of RASi on hemodynamics, left ventricular hypertrophy, and AS progression and prognosis (24, 26–28).

To date, most studies measuring the effects of RASi on the prognosis of AS were non-randomized controlled trials and reported controversial results (2, 13, 14). The meta-analysis by Andersson and Abdulla (29) indicated that treatment with RASi does not increase long-term mortality in patients with AS. The study did not measure the effect of RASi on the prognosis of AS in patients who underwent AVR.

After pooling 3 studies (16–18), our results demonstrated that RASi reduced short-term mortality after TAVR in patients with AS. A similar association was not observed in patients who underwent SAVR. We assume that the discrepancies are largely due to 2 studies included in the SAVR group with a limited sample size (11, 19). Therefore, multicenter randomized controlled trials with large sample sizes are needed to further investigate the effects of RASi on short-term prognosis in patients who undergo SAVR. To date, the effect of RASi on short-term mortality was poorly understood in patients with AS who did not undergo AVR. Although Ardehail et al. (13) reported that RASi did not change short-term mortality (90 days) in patients who did not undergo AVR, the study was non-randomized. In addition, the high proportion of male participants (about 95%) and higher prevalence of hypertension and diabetes mellitus in the RASi group all could affect the results of that study (13). Therefore, future prospective, randomized controlled trials are needed to further explore whether RASi can improve short-term survival in AS patients without indication for AVR.

Consistent with the meta-analysis by Andersson and Abdulla (29), our present study also showed that RASi did not change long-term mortality in patients with AS who did not undergo AVR. Although RASi did not change long-term mortality, previous studies reported that RASi can postpone AS progression and reduce the need for AVR (26–29). Therefore, RASi may be considered in AS patients without indications for AVR. In addition, our meta-analysis of 8 studies (11, 12, 16–21) revealed that RASi reduced long-term mortality in patients who underwent AVR. Subgroup analysis further confirmed that the association persisted in both TAVR and SAVR groups. The reason behind the different effects of RASi on long-term mortality in patients who underwent AVR or not remains unknown. We hypothesize that the contradictory findings are largely due to the following reasons. First, RASi can be associated with the greater regression of left ventricular hypertrophy and fibrosis in patients who underwent AVR compared with those who did not undergo AVR. Second, increased sympathetic activity, which is associated with cardiovascular diseases (30), is more effectively inhibited by RASi in patients who underwent AVR. Third, aortic regurgitation or mitral regurgitation, which is associated with left ventricular remodeling (12, 31, 32), is significantly less severe in patients who underwent AVR. Furthermore, the association between RASi and better clinical outcomes might be mediated by decreasing afterload, valvulo-arterial impedance, systemic vascular resistance, vascular calcification, inflammatory cytokine levels, and endothelial dysfunction (33–36).

### Limitations

There are some limitations in our present study that need to be noted. First, most of the included studies were retrospective cohorts; therefore, our pooled results could be affected by unmeasured confounding variables. Second, due to the heterogeneity of the included trials, a random-effects model was used in the present study. Third, the limited number of studies included in this meta-analysis did not allow us to conduct more subgroup analyses and sensitivity or meta-regression analyses for the outcomes. Finally, the majority of included studies were conducted in highly experienced cardiovascular centers, which could limit the generalizability of our findings to other centers with less expertise.

## Conclusion

The present study demonstrated that RASi was well tolerated in patients with AS. Furthermore, treatment with RASi significantly reduced short- and long-term mortality in AS patients who underwent AVR. To further confirm these findings, large-scale, randomized controlled trials are warranted.

## Data availability statement

The original contributions presented in this study are included in the article/Supplementary material, further inquiries can be directed to the corresponding author.

## Author contributions

HL and QL were responsible for the study conception and design. YG and XK reviewed studies, extracted data, and drafted the manuscript. YG and LZ assessed the quality of the studies included. FG, HZ, and XK performed a systematic search and checked the data. All authors read and approved the final manuscript.
